# Shortest Paths in Multiplex Networks

**DOI:** 10.1038/s41598-017-01655-x

**Published:** 2017-05-12

**Authors:** Saeed Ghariblou, Mostafa Salehi, Matteo Magnani, Mahdi Jalili

**Affiliations:** 1 Faculty of New Sciences and Technologies, University of Tehran, Tehran, Iran; 2 School of Computer Science, Institute for Research in Fundamental Science (IPM), Tehran, Iran; 3 Department of Information Technology, Division of Computing Science, Uppsala University, Uppsala, Sweden; 4 School of Engineering, RMIT University, Melbourne, Australia

## Abstract

The shortest path problem is one of the most fundamental networks optimization problems. Nowadays, individuals interact in extraordinarily numerous ways through their offline and online life (e.g., co-authorship, co-workership, or retweet relation in Twitter). These interactions have two key features. First, they have a heterogeneous nature, and second, they have different strengths that are weighted based on their degree of intimacy, trustworthiness, service exchange or influence among individuals. These networks are known as multiplex networks. To our knowledge, none of the previous shortest path definitions on social interactions have properly reflected these features. In this work, we introduce a new distance measure in multiplex networks based on the concept of Pareto efficiency taking both heterogeneity and weighted nature of relations into account. We then model the problem of finding the whole set of paths as a form of multiple objective decision making and propose an exact algorithm for that. The method is evaluated on five real-world datasets to test the impact of considering weights and multiplexity in the resulting shortest paths. As an application to find the most influential nodes, we redefine the concept of betweenness centrality based on the proposed shortest paths and evaluate it on a real-world dataset from two-layer trade relation among countries between years 2000 and 2015.

## Introduction

Individuals are connected to one other through different interactions such as friendship, conversation, cooperation, and game. Investigating these interactions helps to understand the reason for emergence and development of different societies^1^. Since the pioneering work on group formation by Moreno in 1932 which took the advantages of graphs in order to graphically represent individuals as nodes and the interaction between them as links^2^, this approach has attracted the attention of many researchers^3, 4^. More recently, the heterogeneous nature of social interactions has led to introduce multiplex networks^5–9^ (for the definition of the multiplex and weighted multiplex network refer to Supplementary Note 1). This concept motivated by the fact that many real-world networks are interact with or depend on other networks^10^.

Social ties have different strengths: absent, strong or weak^11^. Interactions also can have positive (e.g., friendship, and collaboration) or negative (e.g., enmity, and hatred) connotation^12^. This differentiation becomes even more significant if the interactions in large-scale Social Network Sites (SNSs) be taken into consideration. Due to the simplicity of creating new relationships in SNSs, most of these relations have weak strengths without emotional closeness and intimacy^13^. Ignoring the links diversity in capacity and intensity and considering all of them as a simple binary relation causes oversimplification and loss of information^14^. This concept motivates many researchers to develop methods for inferring the strength of relations and assign a weight to each link. The weights can correspond to the amount of time which two individuals spend together, services exchange, emotional intensity, the degree of intimacy^11^, trustworthiness^15^, and influence^16^. In this work, we focus only on the influence of the relations as the strength among different nodes (see Supplementary Note 2 for the definition of the influence).

Studies on distance in social networks date back to pioneering works of Simmel^17^ which defined the concept of the *stranger* and Bogardus^18^ which used the concept of *social distance* in measuring prejudice. However, most of the previous efforts on finding the distance between individuals are based on the degree of separation in single-layer networks (without considering the strength of relations and multiplexity)^19, 20^. By considering the influence as weight, finding a path between two individuals that maximizing the influence, will return the indirect influence of individuals upon each other^21^. In multiplex networks, due to the heterogeneity of relations, the concept of the shortest path is different. The heterogeneity in relation types leads to use the concept of Multiple Objective Decision Making (MODM). MODM refers to the process of making decisions in the presence of multiple conflicting objectives (for more information see Supplementary Note 3). In previous work^22^, we introduced a geodesic distance named *Pareto distance* to deal with this heterogeneity, but the weighted nature of relations was ignored in that work which may result in non-optimal paths (for an example of finding optimal paths in multiplex networks in presence and absence of influence of relations refer to Supplementary Note 4). Hence, in this work we introduce a new distance measure based on both the multiplexity and weighted nature of relations, named *influential Pareto distance*. Influential Pareto distance uses the concept of Pareto efficiency and attempts at finding the paths in a multiplex network which have the optimal total weight (e.g., the maximum influence) in each layer separately. We name these paths as *influential Pareto paths*. By taking the advantages of multimodal transportation and edge-colored graph algorithms, we propose an MODM framework and an exact algorithm to find the whole set of these optimal paths. We also redefined the concept of betweenness centrality based on these paths.

## Results

We evaluated our approach on five weighted multiplex datasets from international Trade network^23^, Twitter^24^, Sampson Monastery^25^, Youtube^26^, and StarWars^27^. For more information about the datasets including their density and the total weight of each layer refer to Supplementary Note 5.

### Influential Pareto paths and the usage of different layers

**Figure 1:**
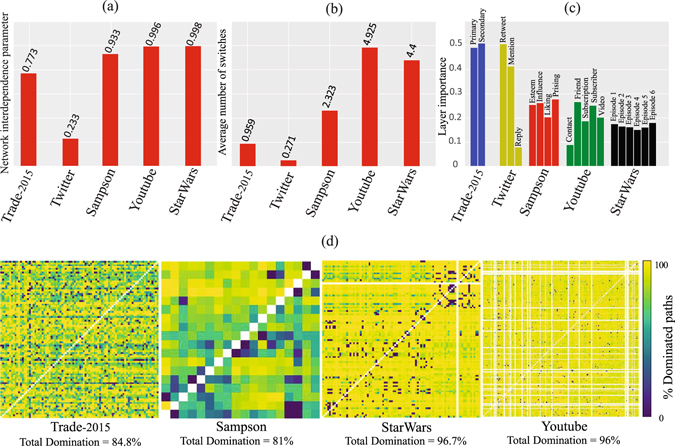
Characteristics of influential Pareto paths. (**a**) Representation of the network interdependence parameter for influential Pareto paths in five datasets. (**b**) Representation of the average number of inter-layer switches. (**c**) The fraction of participation of each layer in the influential Pareto paths. (**d**) Representation of the domination percentage of influential Pareto paths by the paths with minimum number of links traversed in each layer, for each pair of nodes for four datasets; and the total domination percentage of influential Pareto paths. The white points show that there are no paths between two pairs of nodes.

We claim that by considering the influence of relations, the shortest paths will tend to walk through different layers, which means the search will be effective and the individuals will be reachable through using relations in other layers. To support this claim, we use the network interdependence parameter^28, 29^ which is a measure for evaluating node reachability in multiplex networks as follows:

1 $$\lambda =\tfrac{1}{N}\sum _{i\in N}\sum _{\begin{array}{c}j\in N\\ j\ne i\end{array}}\tfrac{{\psi }_{ij}}{{\sigma }_{ij}}$$

where *N* is the number of nodes, *ψ*_*ij*_ is the number of the shortest paths from node *i* to node *j* which use the links in two or more layers, and *σ*_*ij*_ is the total number of the shortest paths from node *i* to node *j*. *λ* lies between zero (when all the links of each shortest paths belong to only one layer) and one (when all the shortest paths use links belong to more than one layers). In this context, we use the influential Pareto paths to obtain the network interdependence parameter for all datasets (see Fig. 1(a)). We also calculate the average number of inter-layer switches for these paths in Fig. 1(b). The results show that all datasets (except for Twitter which has a very low density) have a high value for the network interdependence parameter and the average number of switches. Hence, these influential Pareto paths are compatible with the nature of multiplexity and utilize different layers. One of the inevitable consequences of this compatibility is using these paths in order to find the most influential nodes in multiplex networks.

#### Multiplex influential betweenness centrality

Identifying the main influencers and key players in complex networks has a variety of applications in different fields such as finding epidemic and innovation spreading patterns, cascading failure, and propagation of information^30–32^. Some recent studies on single-layer networks, capitalized on nonbacktracking (NB) matrix^33^ in order to find the influencers. Martin *et al*.^34^ introduced a new centrality measure based on NB matrix to avoid the localization phenomena which arises in eigenvector centrality measure and causes this measure to have a low efficiency. Morone and Makse^35^ mapped the spreading and immunization problems into optimal percolation. Afterward, utilizing a modified NB matrix, they defined the problem of finding the set of optimal influencers as the minimum set of nodes which minimizes the largest eigenvalue of this matrix.

More recently, some studies have focused on finding influential nodes in the existence of different types of relations (i.e., multiplexity)^36, 37^. Pei *et al*.^38^ show that despite the common belief about the importance of social links in information spreading, the effect of other factors and interaction types are also crucial. Hence, in order to find influential nodes for an efficient promotion strategy, different interaction types among individuals must be considered. Domenico *et al*.^36^ redefined some existing centrality measures such as eigenvector and PageRank centralities to work with multiple types of relations, based on the random walk process on these networks. They also defined a path in a multiplex network as a sequence of links starting from a node in layer *x* and ends in a node in layer *y*, and defined a betweenness centrality measure based on this definition of the path. The drawback of this path definition is ignorance of the heterogeneity of the relations and dealing with all links in different layers in the same manner.

**Figure 2:**
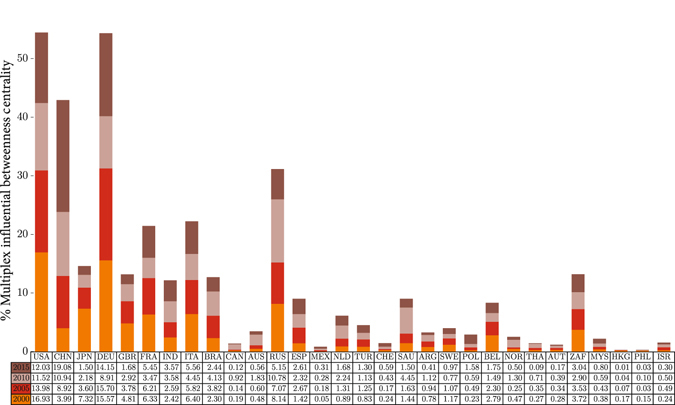
Representation of multiplex influential betweenness centrality for every node in Trade dataset. The bars show the percentage of multiplex influential betweenness centrality of 30 countries for four years. The countries are listed based on their GDP values in 2015 from left to right.

Here we investigate the role of influential Pareto paths on finding the most influential nodes in multiplex networks. For this, we redefine the concept of node betweenness centrality measure. We define the multiplex influential betweenness centrality of node *i* in multiplex network as the number of influential Pareto paths between any two nodes that contains node *i*. Since influential Pareto paths walk through different layers, the nodes with more participation in other layers will have more multiplex influential betweenness centrality and their ranking will be higher. Hence, this measure will present a better ranking for nodes in multiplex network based on their participation in different layers. Figure 2 shows the values of this measure for every node in Trade dataset. The bar charts show this measure for four years (for each year, the percentage of multiplex influential betweenness of each node compared to others is calculated) for 30 countries which have the most Gross Domestic Product (GDP) value. The countries are listed based on their GDP values in 2015 from left to right.

**Figure 3:**
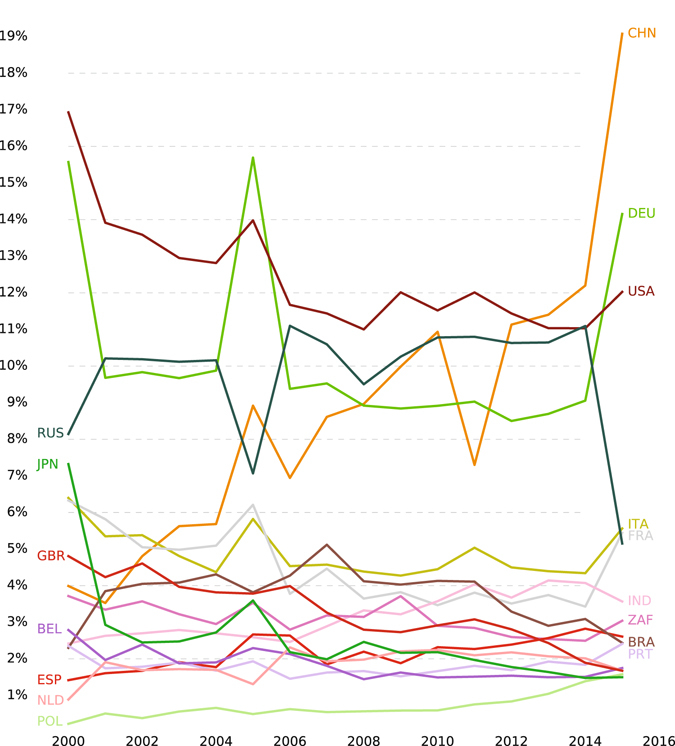
Percentage of multiplex influential betweenness centrality, by country (2000–2015).

Figure 3 shows the importance of countries based on our multiplex influential betweenness centrality measure between the years 2000 and 2015 for fifteen countries which have the most multiplex influential betweenness in the year 2015. This figure shows that there was a significant increment for the importance of China from 2000 to 2015. However, countries like Japan, Great Britain, and the USA has a decrement in their multiplex importance in trade network. Russia also undergoes a significant decrement in its importance in 2014. Some other countries like Italy have a stable ranking from 2000 to 2015.

### Role of weak layers in the shortest paths

The effect of weak layers in multiplex networks first discussed by Lee *et al*.^39^ on cascading failure process in these networks. Weak layers in a multiplex network are those layers with minimal total weights of relations. In order to compute the importance of each layer in optimal paths, we calculate the percentage of participation of each layer in influential Pareto paths based on the percentage of links belonged to each layer. Figure 1(c) shows the importance of layers for five datasets. Our results show that the weak layers play an important role in optimal paths. For example, for the Trade dataset the total weight for secondary industry sector is 9.27 trillion US$ and for primary industry sector is equal to 14.75 trillion US$ which means the secondary industry sector is a weak layer in trade among countries. However, most of the links traversed in influential Pareto paths belongs to this weaker layer. In Sampson dataset, as another example, the weakest layer is Praising layer with the total weight of 77. However, this weak layer plays the most important role in optimal paths and has the highest percentage of participation.

### Influential Pareto paths and the minimum number of links

Another question that requires more attention is “are influential Pareto paths longer than those paths with the minimum number of links traversed in each layer?”. In other words, how many of influential Pareto paths will be dominated by those paths with the minimum number of links traversed in each layer considering only the hop counts. Our results in Fig. 1(d) show that most of the influential Pareto paths (more than 80%) become dominated in different datasets. This means that the influential Pareto paths are not always the shorter ones. The total domination percentage depends on the density of the networks. As it can be seen, for denser datasets (i.e., Trade and Sampson) the total domination percentage is smaller than those with lower density (i.e., StarWars and Youtube). Hence, the influential Pareto paths are more differentiated from shortest paths in low-dense multiplex networks.

## Discussion

In this work, we focus on the problem of finding shortest paths in multiplex networks, a generic term that we use to refer to a number of network models involving multiple types of relationships. While shortest paths in single networks has received considerable attention, this problem in the context of multiplex networks is still a young research area. In current work, we introduced a new distance metric based on the both relations strength and heterogeneity of relations, named influential Pareto distance. We also proposed a multiple objective decision making framework and an exact algorithm to find the whole set of these optimal shortest paths. We name these paths as influential Pareto paths. We evaluate the resulting shortest paths in term of different aspects as network interdependence, average inter-layer switches, role of weak layers, and the length of paths. We also redefined the concept of betweenness centrality based on the influential Pareto paths. Since we observed influential Pareto paths walk through different layers, this new definition presents a better node centrality ranking in multiplex network. Using the proposed metric, we computed the importance of different countries in terms of international trade networks which is a two-layer multiplex network.

Since the problem of finding influential Pareto paths in multiplex networks belongs to the class of NP-completeness, our proposed exact algorithm has limitation for applying to more complex and larger networks. Hence, We proposed a method based on the well-known Nondominated Sorting Genetic Algorithm II (NSGA-II)^40^ framework in order to find the near-optimal solutions in lower time complexity, and evaluated the resulting solutions set of this approach based on different parameters and different performance measures. (see Supplementary Note 7). Some information around the comparision of characteristic of optimal paths in presence and absence of strength of relations has shown in Supplementary Note 6.

## Methods

### Influential Pareto Distance and Influential Pareto Paths

Positive weights need to be maximized through the path and negative weights need to be minimized. The weights can also be multiplicative or additive through the path. However, regardless of minimization or maximization of multiplicative or additive weights, all of such problems can be transformed into a minimization problem of additive weights through the path. Suppose that Θ is a multiplicative metric and we want to find a path *P* from source node *S* to destination node *D* maximizes this metric. Hence, we will have:

$${\rm{\max }}\,\prod _{{x}_{i}\in P}{\rm{\Theta }}({x}_{i})$$

which is equivalent to the following statement (taking the logarithm):

$$\begin{array}{rcl}{\rm{\max }}\sum _{{x}_{i}\in P}\mathrm{log}\,{\rm{\Theta }}({x}_{i}) & = & {\rm{\min }}(-\,\sum _{{x}_{i}\in P}\mathrm{log}\,{\rm{\Theta }}({x}_{i}))\\  & = & {\rm{\min }}\sum _{{x}_{i}\in P}\mathrm{log}\,\tfrac{1}{{\rm{\Theta }}({x}_{i})}\end{array}$$

Thus, in the case of influence, by changing the influence of each link to $log(1/I({x}_{i}))$ (see Supplementary Note 2), the problem will be transformed into the minimization problem of additive weights through the path.

**Definition 1** (***Influential multiplex path length***). *The influential multiplex path length of path p on L networks is defined as a set* (*s*_1_, *s*_2_, …, *s*_*l*_, …, *s*_*L*_), *where s*_*l*_ *is the summation of weights of links traversed in layer l*.

**Definition 2** (***Influential Pareto distance***). *Consider all paths from source node S to destination node D and let IMP*(*S*, *D*) *be the set of all influential multiplex path lengths of these paths*. *The influential Pareto distance from S to D is defined as the set* $SP\subseteq IMP$ *such that* $\forall p\in SP\nexists p^{\prime} \in IMP:p^{\prime} \,\preccurlyeq \,p$.

The notion $\preccurlyeq$ shows the domination relation. The influential Pareto distance corresponds to objective space and is equivalent to Pareto front. Each member of influential Pareto distance can be a map from many paths in decision space. We name the set of all paths in decision space mapped onto influential Pareto distance members in objective space as *influential Pareto paths* set which is equivalent to Pareto set.

### Method for finding influential Pareto paths set in multiplex networks using MODM framework

**Figure 4:**
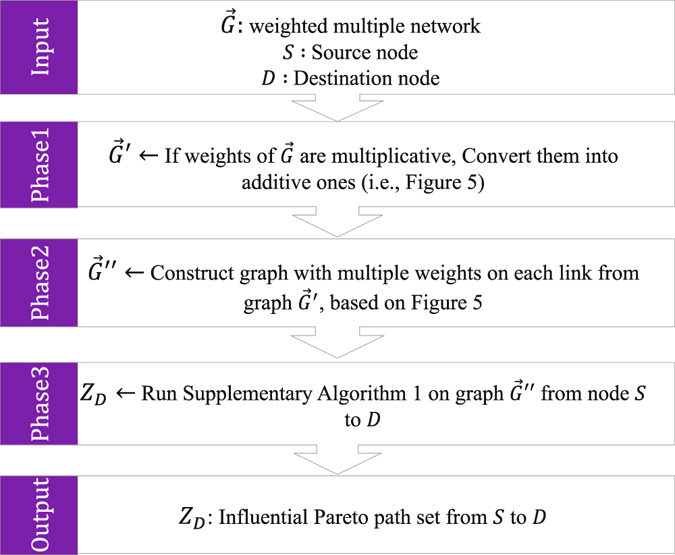
Different phases of our proposed method in order to find influential Pareto path set in weighted multiplex networks.

**Figure 5:**
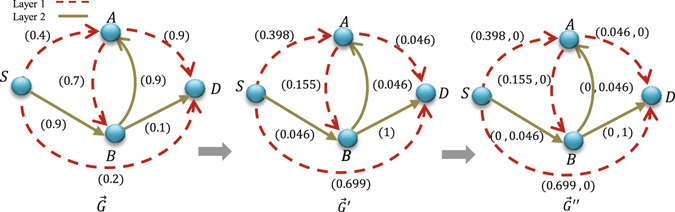
Construction of graph $\vec{G}^{\prime\prime}$ for the two-layer weighted multiplex network. $\vec{G}$ is a two-layer multiplex network with influence on each link. $\vec{G}^{\prime}$ is the graph constructed by changing the influence of each link based on $log(1/I({x}_{i}))$ in order to transform the problem onto the problem of minimization of additive weights. $\vec{G}^{\prime\prime}$ is the multiplex network with multiple weights on each link constructed from $\vec{G}^{\prime}$.

Here, we propose a method for finding the whole set of influential Pareto paths. For this, we consider three main phases according to Fig. 4. The phase 2, contains two steps as follows:

1. We ascribe to each link in the multiplex network a weight vector ${W}_{{(ij)}^{\gamma }}=({w}_{{(ij)}^{\gamma }}^{1},{w}_{{(ij)}^{\gamma }}^{2},\ldots ,{w}_{{(ij)}^{\gamma }}^{L})$ where *L* is the number of layers, and construct a new multiplex network $\vec{G}^{\prime\prime}$.
2. For each link in network $\vec{G}^{\prime\prime}$, if it belongs to layer *l*, we set *l*-th weight of the link equals to the weight of the link in $\vec{G}^{\prime}$ and set all the other weights of that weight vector equals to zero (Fig. 5).

At phase three, Supplementary Algorithm 1 will apply to the graph $\vec{G}^{\prime\prime}$. This algorithm will find the set of multiobjective shortest paths (MOSP) from *S* to *D* in $\vec{G}^{\prime\prime}$, which is itself the influential Pareto path set for graph $\vec{G}^{\prime}$. Afterward, the influential Pareto distance set can simply infer from this set. Since most of the algorithms on finding MOSP work on single-layer networks, we improve one of the existing exact algorithms of MOSP in single-layer networks to work with multiplex networks.

MOSP algorithms in single-layer networks can be restricted to four following species: exact, heuristic, approximate, and meta-heuristic^41–44^. Many of these algorithms (especially heuristic and approximate ones) assume that the weights are nonzero. Hence they cannot be generalized for our problem which most of the weights are zero. In order to find MOSP in graph $\vec{G}^{\prime\prime}$, we improved an exact label-setting algorithm presented by Martin and Santos^45^ to works with different types of links. The mathematical formulation of the problem is as follow:

Suppose that we construct the multiplex graph $\vec{G}^{\prime\prime}$ with a vector of weights ${W}_{{(ij)}^{\gamma }}=({w}_{{(ij)}^{\gamma }}^{1},{w}_{{(ij)}^{\gamma }}^{2},\ldots ,{w}_{{(ij)}^{\gamma }}^{L})$ on each link. Assume that *X* is a vector consisting of ${x}_{ij}^{\gamma }$, where ${x}_{ij}^{\gamma }=1$ indicates that (*i*, *j*)^*γ*^ is a link on the path and ${x}_{ij}^{\gamma }=0$ otherwise. Then a path is composed of the links with ${x}_{ij}^{\gamma }=1$. The problem of finding multiobjective shortest path from *S* to *D* in multiplex network $\vec{G}^{\prime\prime}$ can be formulated as follows:

$$\begin{array}{c}minF(x)=\{\begin{array}{c}\begin{array}{c}{f}_{1}(x)=\sum _{\gamma \in (1,\ldots ,L)}\sum _{{(i,j)}^{\gamma }\in {E}_{\gamma }}{w}_{{(i,j)}^{\gamma }}^{1}{x}_{ij}^{\gamma }\\ {f}_{2}(x)=\sum _{\gamma \in (1,\ldots ,L)}\sum _{{(i,j)}^{\gamma }\in {E}_{\gamma }}{w}_{{(i,j)}^{\gamma }}^{2}{x}_{ij}^{\gamma }\\ \vdots \\ {f}_{L}(x)=\sum _{\gamma \in (1,\ldots ,L)}\sum _{{(i,j)}^{\gamma }\in {E}_{\gamma }}{w}_{{(i,j)}^{\gamma }}^{L}{x}_{ij}^{\gamma }\end{array}\end{array}\end{array}$$

Subject to

$$\sum _{\gamma \in (1,\ldots ,L)}\sum _{j:{(i,j)}^{\gamma }\in {E}_{\gamma }}{x}_{ij}^{\gamma }-\sum _{\gamma \in (1,\ldots ,L)}\sum _{j:(j,i{)}^{\gamma }\in {E}_{\gamma }}{x}_{ji}^{\gamma }=\{\begin{array}{c}\begin{array}{cc}1 & {\rm{i}}{\rm{f}}i=S\\ 0 & {\rm{i}}{\rm{f}}i\ne S,D\\ -1 & {\rm{i}}{\rm{f}}i=D\end{array}\end{array}$$

$${x}_{ij}^{\gamma }\in \{0,1\},\,{(i,j)}^{\gamma }\in {E}_{\gamma },\gamma \in (1,\ldots ,L)$$

This problem belongs to the class of multiobjective combinatorial optimization (MOCO) and is NP-complete. The pseudocode of our extension of *Martin and Santos* exact algorithm using the notion from Garroppo *et al*.^46^ described in Supplementary Note 8.

## Electronic supplementary material

Supplementary Information
