# Supplementary material for: Shortest Paths in Multiplex Networks
Source: Sci Rep. 2017 May 12;7:2142. doi: 10.1038/s41598-017-01655-x (PMC5438413; doi:10.1038/s41598-017-01655-x)
Supplement: Supplementary file 1 — Supplementary Information [file 41598_2017_1655_MOESM1_ESM.pdf]

Manuscript ID: SREP-17-00953-A
Title: Shortest Paths in Multiplex Networks
Authors: Saeed Ghariblou , Mostafa Salehi, Matteo Magnani and Mahdi Jalili

---

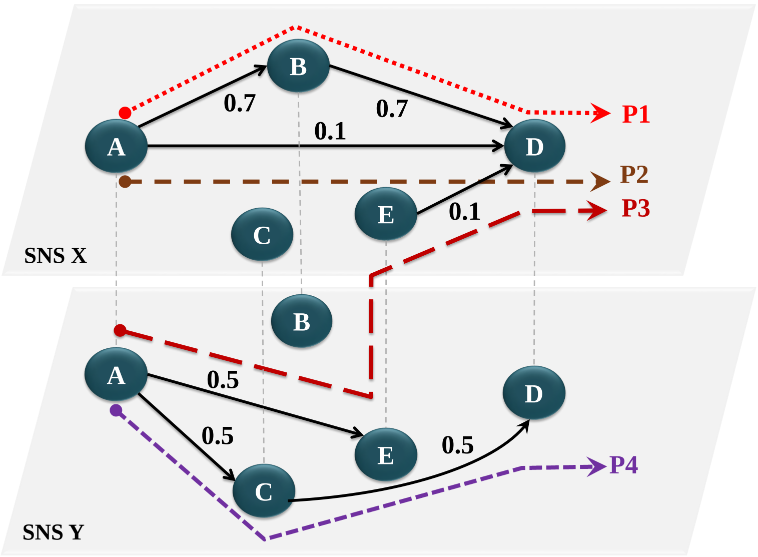

	Link Count		Influence	
**Paths**	**SNS X**	**SNS Y**	**SNS X**	**SNS Y**
**P1**	**2**	**0**	**0.49**	**1**
**P2**	**1**	**0**	**0.1**	**1**
**P3**	**1**	**1**	**0.1**	**0.5**
**P4**	**0**	**2**	**1**	**0.25**

Supplementary Figure 1. Representation of a multiplex network consisting of relations between individuals in two different social network Sites X and Y. The weight of each link shows the direct influence from one person to another in that SNS. If we ask person A to introduce us to person D for a job employment, with considering relations in both SNSs there will be four possible paths for this introduction. The table shows the number of links traversed in each layer for these four paths and the influence of these paths in each layer. The question that arises here is that which one of these paths is more optimal and will increase the probability of this employment.

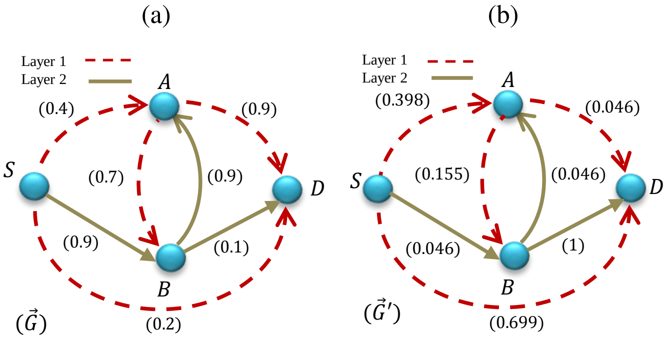

(c)

Paths	Multiplex Path Length		influential Multiplex Path Length	
	L1	L2	L1	L2
**P1**: $((S,A)^1,(A,D)^1)$	2	0	0.444	0
**P2**: $((S,A)^1,(A,B)^1,(B,D)^2)$	2	1	0.553	1
**P3**: $((S,B)^2,(B,A)^2,(A,D)^1)$	1	2	0.092	0.046
**P4**: $((S,B)^2,(B,D)^2)$	0	2	0	1.046
**P5**: $((S,D)^1)$	1	0	0.699	0
	**Pareto Distance**		**influential Pareto Distance**	
	0	2	0.444	0
	1	0	0.092	0.046
			0	1.046

Supplementary Figure 2. (a) A two layer multiplex network $\vec{G}$ with the influence of links. Each layer is represented using a different color. Links have weight based on their influence. There are five paths from S to D (ignoring loops). (b) Transforming the problem of maximizing multiplicative weights into the problem of minimizing additive weights through the paths with changing the weights of each link x_i to $log(^1/_{I(x_i)})$ and construction of multiplex network $\vec{G}'$. (c) Representation of multiplex path length, influential multiplex path length, Pareto distance and influential Pareto distance for paths from S to D. Each path has a multiplex path length and an influential multiplex path length. Pareto distance set has two members corresponds to paths $P4$ and $P5$ (i.e., Pareto path set) which means these two paths have the minimum number of links traversed in each layer. Influential Pareto distance set has three members corresponds to paths $P1$, $P3$ and $P4$ (i.e., influential Pareto paths set) which means these paths are more influential in each layer rather than other paths. These differences in resulting paths show that first of all, paths with a minimum number of links in each layer may not be a good choice for purposes such as sending messages. Since the path $P5$ has the minimum links traversed in each layer, however, by considering the influence of relations, it will be dominated by other paths which mean it has a weak strength and is less influential than other paths. Secondly, paths which have not a minimum number of links traversed in each layer (i.e., dominated by the other paths), might be better paths for sending messages. Since in this exampl,e the path $p3$ is dominated by other paths with respect to the number of links traversed in each layer but considering the strength of relations, it will not be dominated by other paths which mean it has a strong strength and is more influential than the others.

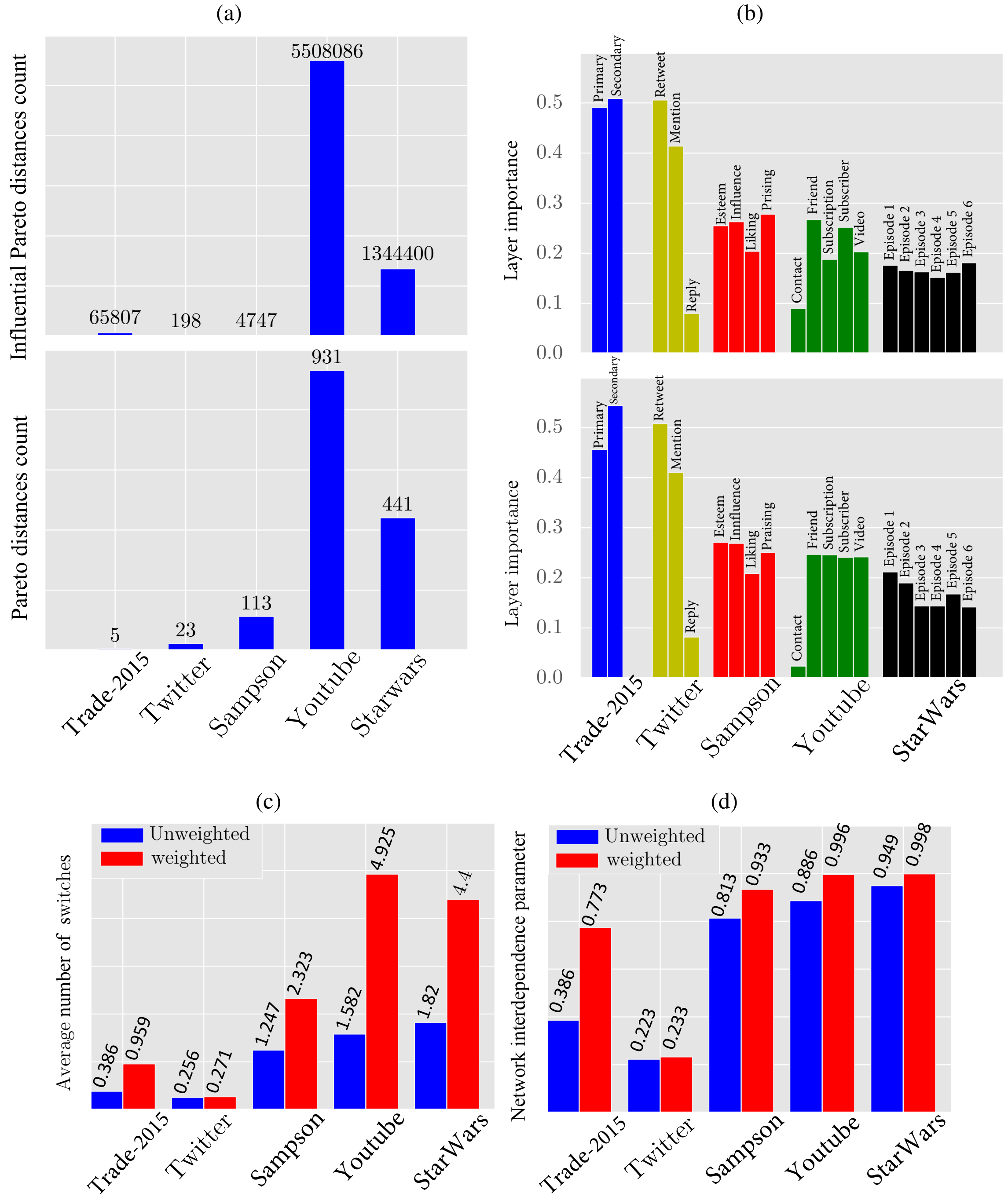

Supplementary Figure 3. (a) Comparision of the number of Pareto distances and influential Pareto distances. (b) Representation of the importance of different layers in Pareto and influential Pareto paths. (C) Representation of the average number of inter-layer switches. (d) Representation of the network interdependence parameter.

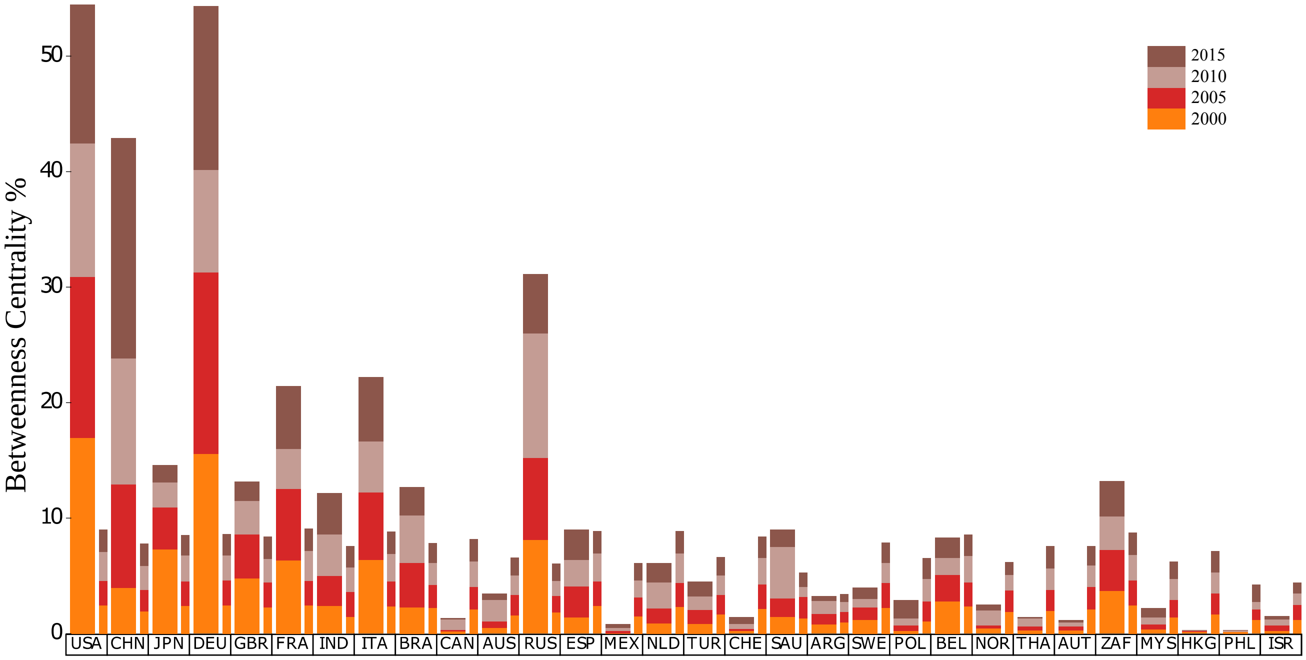

Supplementary Figure 4. Representation of comparison of multiplex betweenness and influential multiplex betweenness centrality, for every node in Trade dataset. The bars show the percentage of multiplex betweenness (thin bars) and influential multiplex betweenness centralities (thick bars) for four years for 30 countries with higher GDP values in 2015. The countries are listed based on their GDP values in 2015 from left to right.

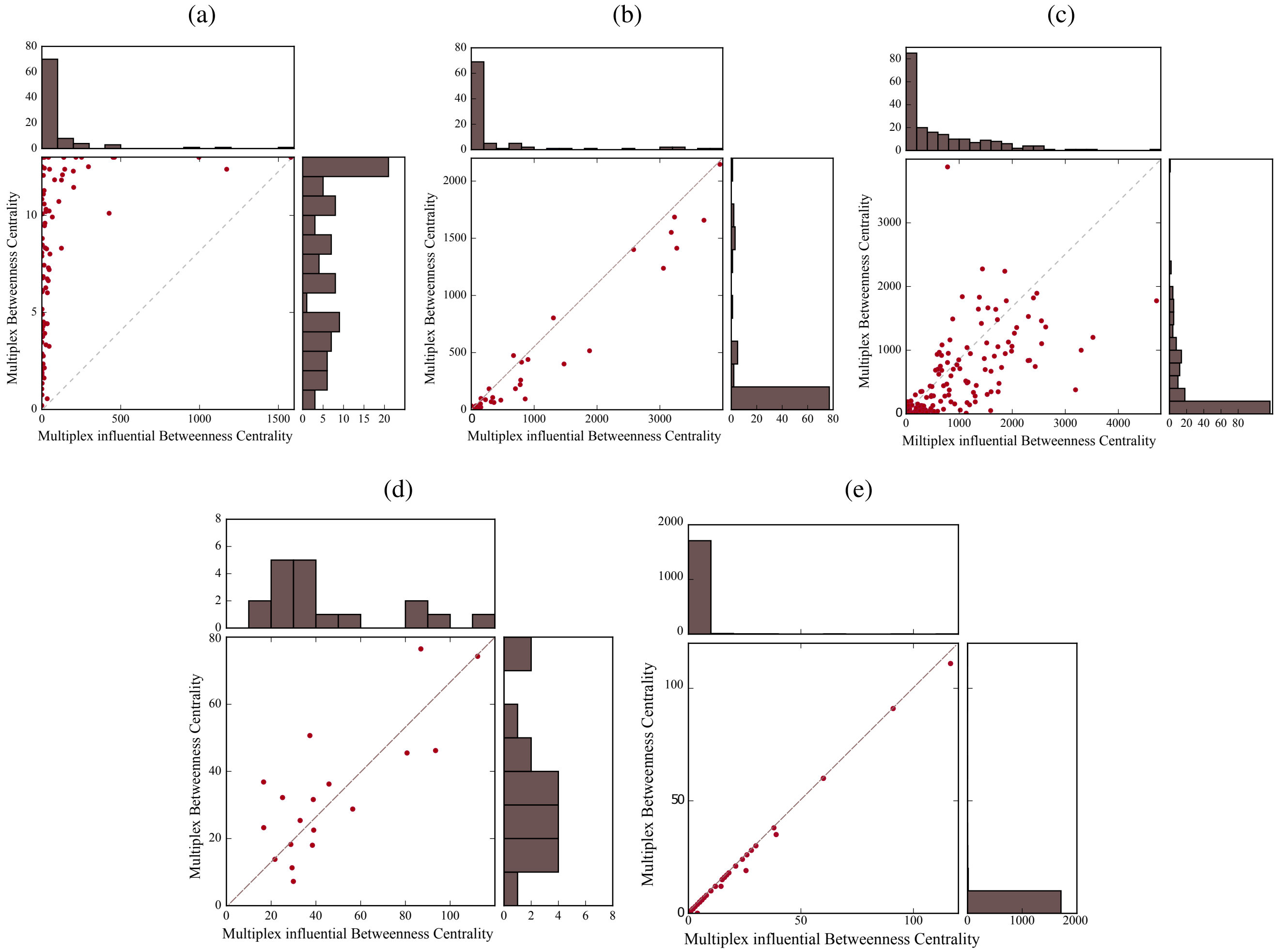

Supplementary Figure 5. (a) Representation of the difference between ranking of nodes based on multiplex influential betweenness centrality and multiplex betweenness centrality for five different datasets: (a) Trade network for the year 2015 (b) StarWars (c) Youtube (d) Sampson (e) Twitter.

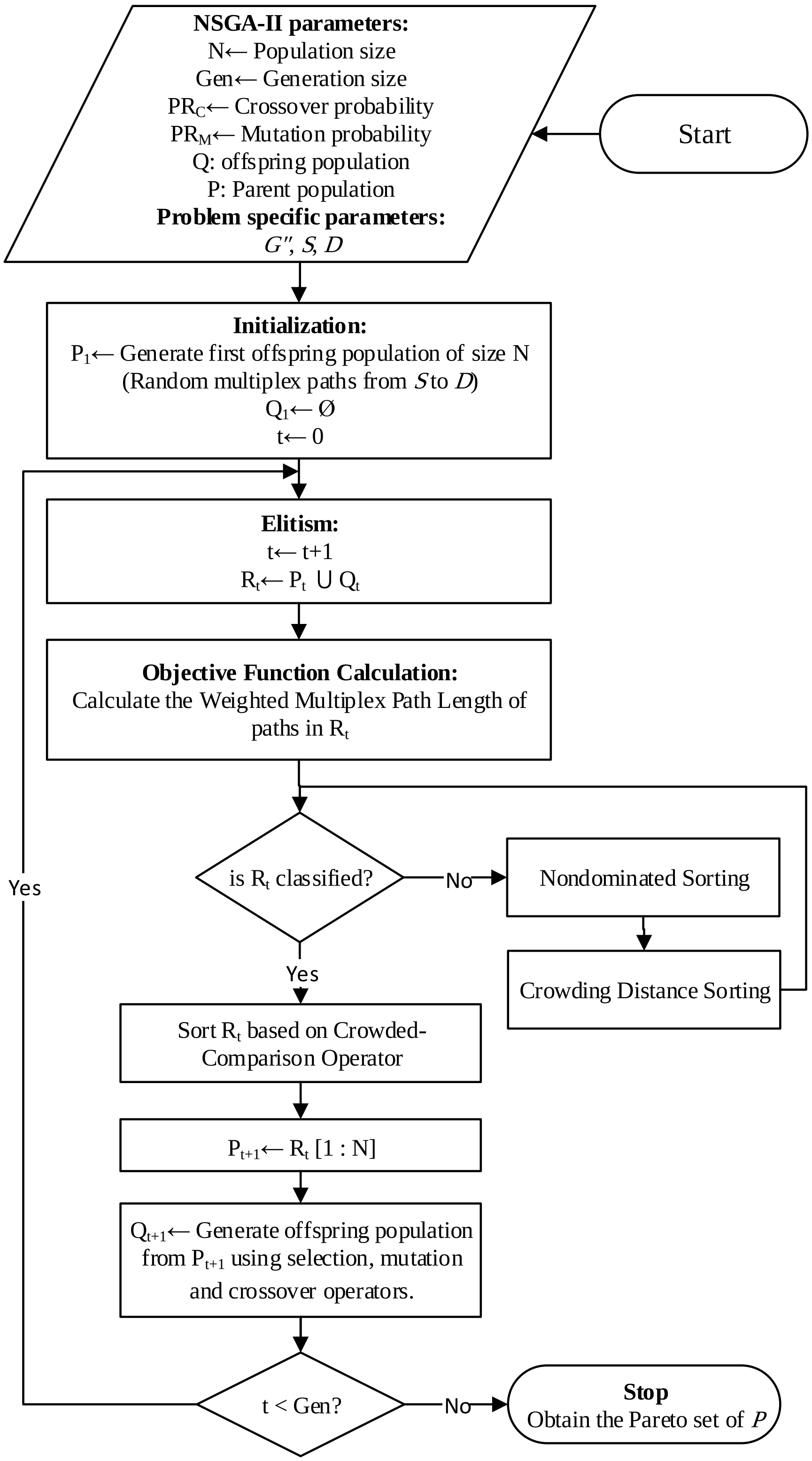

Supplementary Figure 6. Flowchart of NSGA-II procedure for finding approximate set of influential Pareto paths in multiplex networks.

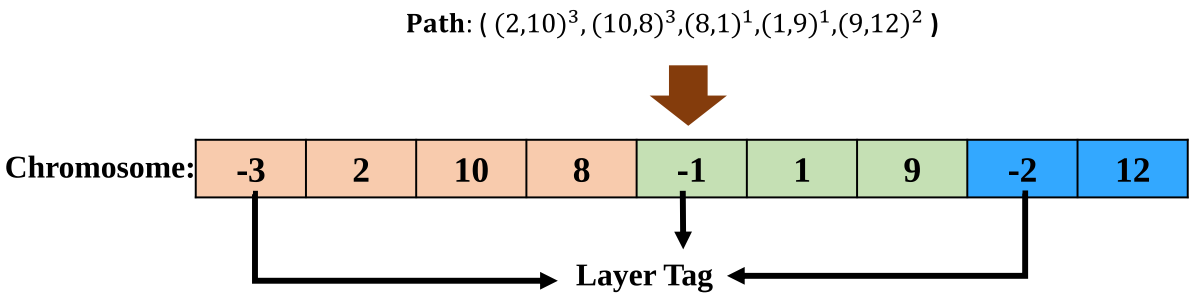

Supplementary Figure 7. An example of the encoding method for a multiplex path.

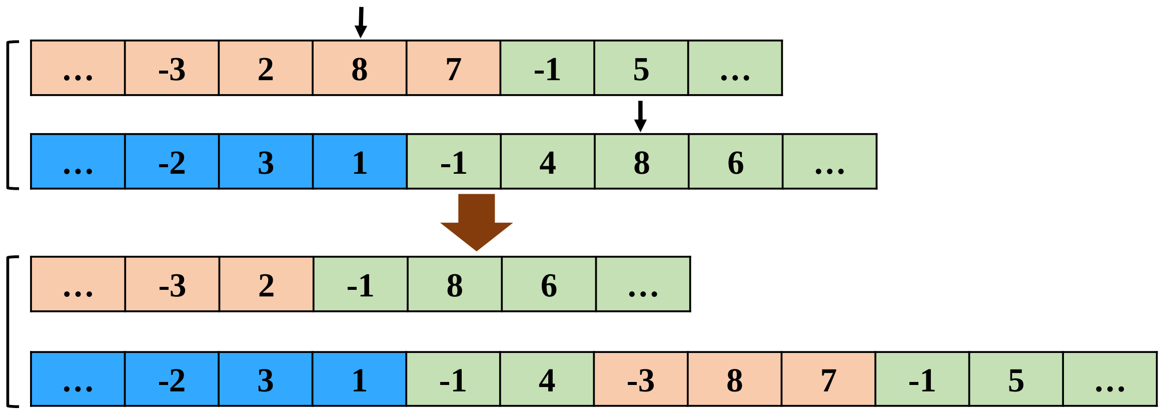

Supplementary Figure 8. Crossover operator on two multiplex paths.

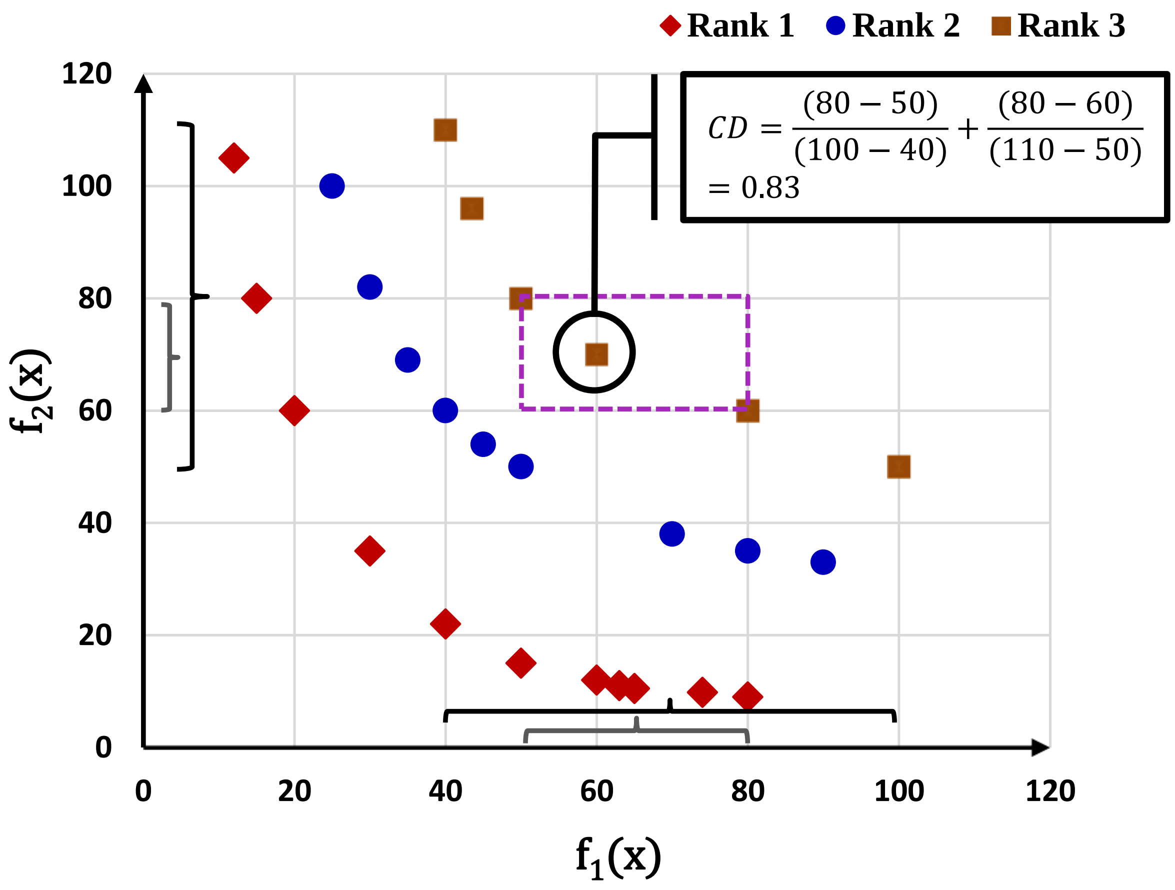

Supplementary Figure 9. Classification of population in NSGA-II procedure based on nondominated sorting and crowding distance.

(a) The effect of increasing crossover rate

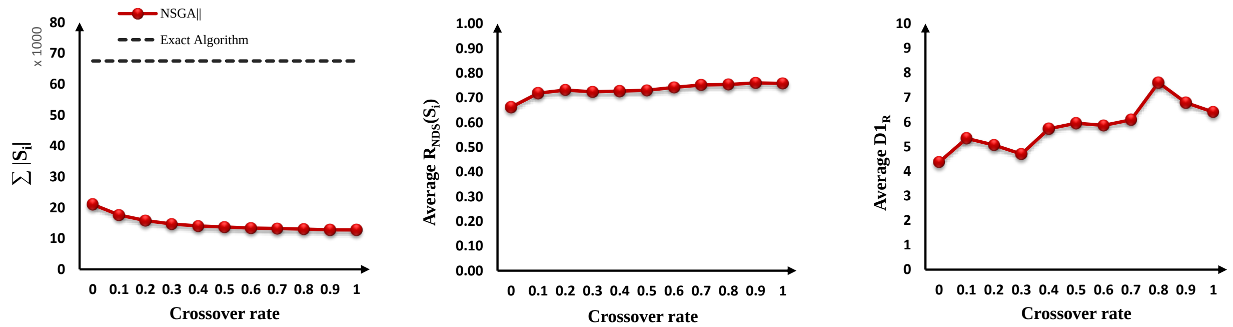

(b) The effect of increasing mutation rate

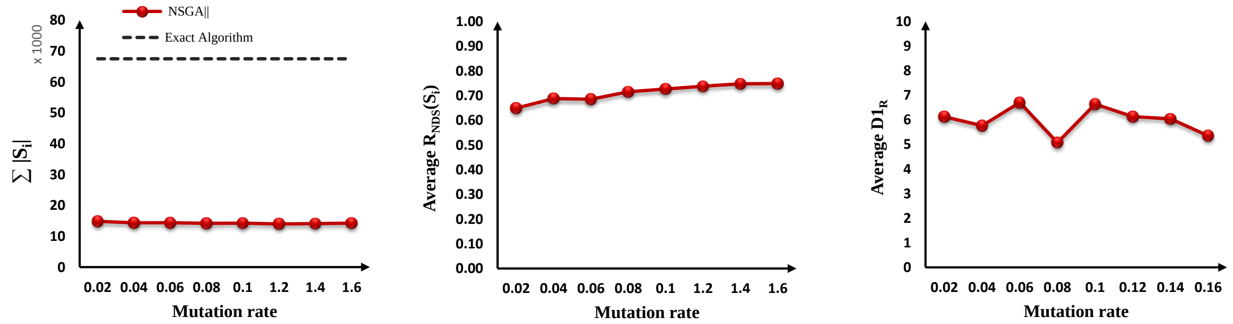

(c) The effect of increasing generation size

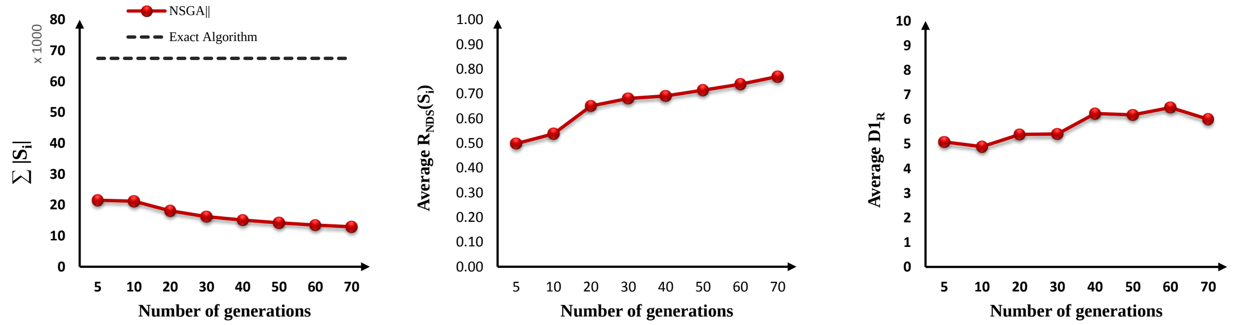

(d) The effect of increasing population size

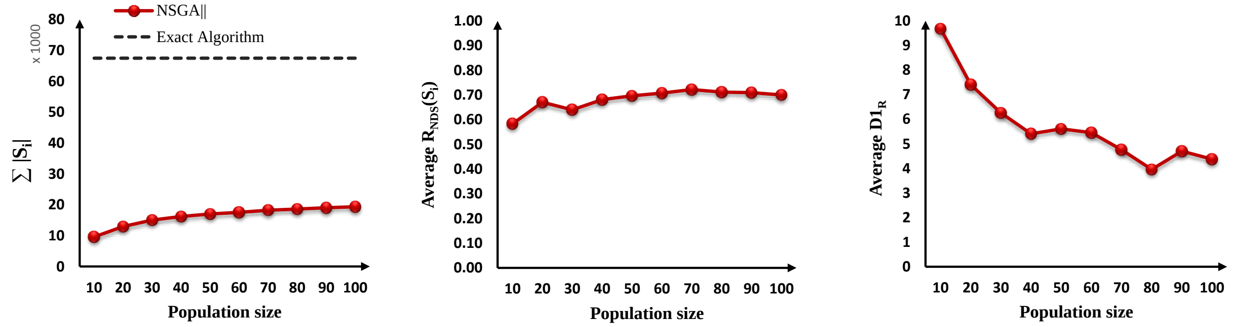

Supplementary Figure 10. The effect of varying the values of different parameters of NSGA-II in performance measures of this algorithm for the international Trade network of 2015.

		Number of active nodes	Number of links	Network density	Total weight
Trade (2015)	Primary	88	7236	0.945	14.75 tUS$
	Secondary	88	6998	0.914	9.27 tUS$
Youtube	Contact	18	12	0.0006	12
	Friend	116	323	0.016	866
	Subscription	125	863	0.043	1259
	Subscriber	111	371	0.019	704
	Video	119	605	0.0006	2023
Sampson	Esteem	18	54	0.176	107
	Influence	18	53	0.173	106
	Liking	18	56	0.183	111
	Praising	18	39	0.127	77
Twitter	Retweet	1167	910	0.0003	1133
	Mention	760	531	0.0002	653
	Reply	239	134	0.00004	155
StarWars	Episode1	38	148	0.035	503
	Episode2	33	103	0.024	215
	Episode3	24	67	0.016	225
	Episode4	21	61	0.014	299
	Episode5	21	59	0.014	309
	Episode6	20	60	0.014	293

Supplementary Table 1. The characteristics of five different weighted multiplex datasets based on their number of active nodes, the number of links, the network density and the total weights of relations in each layer. tUS$ stands for trillion United Stated dollars.

1 Supplementary Note 1 (Definition of multiplex and weighted multiplex network)

A multiplex network can be formally represented as a vector of networks $\vec{G} = (G_1, G_2, ..., G_\gamma, ..., G_L)$, where $\{G_\gamma\}_{\gamma=1}^{L} = \{(V, E_\gamma)\}_{\gamma=1}^{L}$. L shows the number of networks (i.e., layers), V is the set of N nodes which is the same for all layers, and E_γ is the set of links correspond to layer γ. We show each link with $(i,j)^\gamma$ which defines a link from node i to node j in layer γ. By ascribing weight ($w_{(ij)^\gamma}$) to each link $(i,j)^\gamma$, the weighted multiplex network will be constructed[1]. In the context of social networks, nodes represent individuals and each layer represents a different type of social relations.

2 Supplementary Note 2 (The concept of influence)

The importance of social influence on applications like viral marketing, item recommendation, information propagation and link prediction leads most of the attentions toward inferring the *influence* of ties based on topic based models[2], links information[3] or a combination of both methods[4]. In this work, we use the *influence* as weights of relations based on the definition of Hangal *et al.*[3] which models the direct influence of individual i on j proportional to the investment of j over i as follows:

$$I(i,j) = \frac{invest(j,i)}{\sum_{x \in V} invest(j,x)} \tag{1}$$

This investment can be anything such as the amount of time that individuals spend together or the number of papers they coauthor. As an example in the Twitter SNS, the influence of A on B can be considered as the number of times that B retweets A divided by the total retweets of B. Based on this definition, influence is asymmetric (i.e., if individual A has an influence on B, it does not mean that B has an influence on A as well) and ranges from 0 to 1.

3 Supplementary Note 3 (Multiple Objective Decision Making)

Multiple objective decision making (MODM) is a branch of multiple criteria decision making (MCDM) and refers to the process of decision making in the presence of multiple conflicting criteria where the decision space is continuous. Because of the nature of MODM problems, they primarily indicate using mathematical programming with a set of multiple objective functions and a set of well-defined constraints[5,6]. The general formulation of MODM problem for when decision maker wants to minimize the objective functions is as follows:

$$\begin{aligned} \min \quad & F(x) = [f_1(x), f_2(x), ..., f_K(x)] \\ \text{s.t.} \quad & x \in \chi_f \end{aligned} \tag{2}$$

Where x is an n-dimensional vector of decision space variables, K is the number of objective functions and χ_f is the set of all feasible solutions. Based on the types of the objective functions, the decision space variables, and the constraints of the problem, different forms of mathematical programming appears.

Since modeling of many real-world problems involve discrete representation using integer variables, Multiple Objective Integer Programming (MOIP) has been increasingly studied in recent years. In MOIP problem, we have $\{f_i(x) = C^T x : i \in \{1,2,...,K\}\}$, where C is an $1 \times n$ vector in $\mathbb{Z}$ and $\chi_f = \{x \in R^n \mid Ax = b, x \geq 0, x \in \mathbb{Z}\}$, where A is an $m \times n$ constraint matrix. A special case of MOIP where the set of constraints is in specific and structural form, and decision space variables are particularly a subset of the set $\{0,1\}^n$, is known as multiple objective combinatorial optimization (MOCO) which comprises multiple objective

version of many well-known classical problems such as shortest path, minimum spanning tree, traveling salesman, knapsack, and assignment[7,8]. MOCO problems belong to the class of NP-completeness and have three main difficulties: the largeness of efficient solution set, the non-convexity of feasible solution set (these two are common difficulties with MOIP problems), and inability to introduce additional constraints during solving process (this one is specific to MOCO problem)[9].

Finding efficient solutions in MODM problems leads to the concept of Pareto optimality. Pareto optimal solutions are those for which improving in one objective cannot occur without the worsening of at least one other objective. Understanding the concept of Pareto optimal solution requires knowledge around the partial order relation called *dominance*. For two feasible solution x and y, we say x dominates y, or y is dominated by x if the objective functions respect the following condition:

$$f(x) \neq f(y) \quad \wedge \quad f_k(x) \leq f_k(y) \qquad \forall k \in [1,2,...,K] \tag{3}$$

This relation is denoted by $x \prec y$. The notion $x \preceq y$ also use for *dominates or equals* relation. For the feasible solution x, if no other feasible solution dominates x we say that x is a *nondominated* solution or a *Pareto optimal* solution. Based on the type of the problem, more often there is more than one Pareto optimal solution. In some problems such as multiobjective shortest path (MOSP), it is proven that the number of Pareto optimal solutions grow exponentially with the number of nodes of the input network and the problem is NP-complete[10]. The set of all Pareto optimal solutions known as *Pareto set* (*PS*) as follows:

$$PS = \{x \in \chi_f \mid \nexists y \in \chi_f : f(y) \prec f(x)\} \tag{4}$$

The representation of Pareto set in the objective space known as *Pareto front* (PF) as follows:

$$PF = \{F(x) = (f_1(x),...,f_m(x)) \mid x \in PS\} \tag{5}$$

Note that Pareto set refers to decision variable space and Pareto front refers to objective space.

4 Supplementary Note 4 (An example of the effects of the influence on shortest paths in multiplex networks)

Consider interaction of individuals in two different Social Network Sites (SNSs) X and Y according to Supplementary Figure 1. In each SNS, weights represent the direct influence between two individuals in that social network. Suppose that we ask person A to introduce us to person D for job employment (or send our message to person D). What is the optimal path from A to D? Considering only network Y there will be no path for this aim. Taking both networks into account (i.e., multiplexity) it is possible through four paths $p1$ (i.e., A ask B to introduce us to D), $p2$, $p3$, and $p4$. Consider two different cases; when the influence of individuals on each other are ignored, and when the networks are weighted based on the degree of influence of individuals upon each other. In the former case, the optimal path will be the one with the minimum number of links (i.e., hop counts). In the latter case, the optimal path will be the one with the maximum path influence (*path influence* of path p equals to the product of the influence of each link on the path). Due to the heterogeneity of relation types, the links in different layers can not be combined in order to find the optimal path and are needed to be optimized separately[11].

In case one, length of the paths will be $p1 = (2X,0Y)$, $p2 = (1X,0Y)$, $p3 = (1X,1Y)$, and $p4 = (0X,2Y)$, where $p = (iX, jY)$ means the path p has i links traversed in layer X and j links traversed in layer Y. Undoubtedly, path $p2$ is better than $p1$ since the number of links traversed in each layer for this

path is less than $p1$ (i.e., $p2$ dominates $p1$. The *domination* relation is explained in Supplementary Note 3). $p2$ also dominates $p3$. Comparing the paths $p2$ and $p4$, since the number of links traversed for $p2$ in one layer is more and in another layer is less than the number of links traversed by $p4$, therefore these paths are incomparable. In this case, deciding on which path is better involves a prior knowledge around the importance of different layers. Since there is no such knowledge, more than one solution can exist. Here, both $p2$ and $p4$ are the optimal paths. At the next step, the decision maker (DM) may choose the best path from these solutions.

In the second case, the influence of the paths will be $p1 = (0.49X, 1Y)$, $p2 = (0.1X, 1Y)$, $p3 = (0.1X, 0.5Y)$, and $p4 = (1X, 0.25Y)$, where $p = (iX, jY)$ means the path p has i influence in layer X and j influence in layer Y. Here $p1$ will be better than $p2$ since the influence of the link $(A,D)^X$ is very weak and D will not accept the request of A (this could happen in situation where a person is a hub, like celebrities and politicians with many friends which none of them have influence on him). Hence, since A has a high influence on B and B has a high influence on D, path $p1$ will be a better option for this introduction, although it has more links than $p2$. In this case, $p1$ and $p4$ are the optimal paths (we name these paths as the influential Pareto paths). As a consequence, ignorance of the influence of relations may cause in non-optimal paths.

5 Supplementary Note 5 (Datasets)

We evaluated our approach on five weighted multiplex datasets as follows:

- **Trade dataset**: Contains the trade relations among countries for each year between 2000 and 2015, and is a two-layer multiplex network of trade relations in the primary and secondary industries. This data has been obtained from UN COMTRADE database[12] which contains the trade relations among countries based on specific commodities for each year since 1962. We construct a two-layer multiplex network from this database containing the trade relations in primary and secondary industries based on the work by Lee *et al.*[13]. We used the SITC Rev.2 commodity classification and considered the classification codes from 0 to 4 as the primary industry (Layer 1) and the classification codes from 5 to 8 as the secondary industry (Layer 2). The weights of the relations show the trade volume based on US dollars. We only consider the countries in which their trade information existed in UN COMTRADE database for all years from 2000 to 2015 and their Gross Domestic Product (GDP) data for the year 2015 existed in the World Economic Outlook (WEO) database[14]. There was 88 country with mentioned features.

- **Twitter dataset**: This dataset is a three-layer weighted multiplex network, with layers correspond to *retweets*, *mentions*, and *replies* relations in Twitter SNS collected by Omodei *et al.*[15] during the Cannes Film Festival in 2013. We uniformly at random selected 20000 nodes from this dataset with all of their connected links as a sampled dataset. Since many of nodes were isolated in all layers, we removed these Nodes. The remaining network was a three layer weighted multiplex network with 1734 nodes.

- **Sampson Monastery dataset**: This dataset is an eight-layer weighted multiplex network. The layers correspond to the social relation among 18 individuals who were preparing to enter a monastery[16]. From this dataset we only consider positive relations which include four layers corresponding to *esteem*, *influence*, *liking*, and *praising* relations. the weights show the intensity of these relations.

- **Youtube dataset**: This dataset is a five-layer weighted multiplex dataset of different interactions between users in Youtube video sharing site collected by Tang *et al.*[17] in 2008. We uniformly at

random selected 200 nodes from this dataset with all of their connected links as the sampled dataset. The five layers correspond to contact network between users, the number of shared friends, number of shared subscriptions, number of shared subscribers, and number of shared favorite videos among users.

- **StarWars dataset**: This dataset is a six-layer weighted multiplex network of 92 characters of StarWars movies. each layer corresponds to an episode and the links between characters are based on the number of times that the individuals mentioned in the same scene[18].

Supplementary Table 1 shows the detail information about these datasets.

6 Supplementary Note 6 (Optimal paths in weighted and unweighted multiplex networks)

Without considering the weights of relations, the shortest paths in multiplex network will be those which have the minimum number of links traversed in each layer separately. In previous work[11], we introduced a geodesic distance named Pareto distance in order to deal with the heterogeneity of relation types in multiplex networks. In the following, we bring some definitions on shortest paths in unweighted multiplex networks and compare the shortest paths in weighted and unweighted multiplex networks.

Definition 1 *(**Multiplex Path Length**). The multiplex path Length of path p on L networks is defined as a set* $(r_1, r_2, ..., r_l, ..., r_L)$*, where* r_l *is the number of links traversed in layer l.*

Definition 2 *(**Pareto Distance**). Consider all paths from source node S to destination node D in a multiplex network, and let* $MP(S,D)$ *be the set of all multiplex path lengths of these paths (possibly fewer in numbers since several paths might have the same multiplex path length). The Pareto Distance from S to D is defined as the set* $P \subseteq MP$ *such that* $\forall p \in P \nexists p' \in MP : p' \preceq p$*.*

The Pareto distance corresponds to objective space and is equivalent to Pareto front. Each member of Pareto distance can be a map from many paths in decision space. We name the set of all paths in decision space mapped onto Pareto distance members in objective space as *Pareto path* set which is equivalent to Pareto set. Supplementary Figure 2 shows an example of comparison between multiplex path length, influential multiplex path length, Pareto distance and influential Pareto distance.

Our results in Supplementary Figure 3(a) show that the number of Pareto distances and influential Pareto distances depends on the density of the network, and the number of layers. Our results also show that the number of influential Pareto distances are much higher than Pareto distances. In the context of the importance of layers, there are no significant differences in Pareto and influential Pareto paths (Supplementary Figure 3(b)). For the number of switches (Supplementary Figure 3(c)) and the network interdependence parameter (Supplementary Figure 3(d)), influential Pareto paths have higher values compared with Pareto paths. This means that influential Pareto paths are more tended to utilize the different layers compared to Pareto paths. Hence, they will be a better indication of the importance of nodes in multiplex networks. In our previous wok[19], we defined the multiplex betweenness centrality of node *i* as the number of Pareto paths between any two nodes that contains node *i*. Supplementary Figure 4 shows a comparison of multiplex betweenness and influential multiplex betweenness centralities, for every node in Trade dataset. This figure emphasizes the significant difference in node ranking for the two measures. Based on multiplex betweenness, France has the highest total ranking in four years among countries, but our multiplex influential betweenness infers the US as the highest total ranking. Supplementary Figure 5 present the difference in values of these two multiplex betweenness centrality measures and their correlation for all five datasets.

7 Supplementary Note 7 (Using NSGA-II for finding near-optimal solution set)

Supplementary Figure 6 shows the flowchart of NSGA-II procedure for our problem of finding the influential Pareto paths in multiplex networks. The main steps of this flowchart are explained in the following.

7.1 Encoding method and initial population

Due to the existence of multiple layers, a multiplex path cannot be represented only by its sequence of nodes. Hence, in order to represent a path in a multiplex network as a sequence of genes, we utilize the encoding method represented by Yu & Lu[20]. Based on this encoding method, a chromosome consists of a number of negative integers (representing layers tag) followed by a number of positive integers (representing node IDs). Figure 7 shows an example of a multiplex path and its correspondent encoding chromosome which can handle the existence of multiple layers.

In order to generate the initial population of N multiplex paths from source node S to destination node D in a multiplex network, we utilize the depth-first search (DFS) algorithm with two modifications:

1. At each node i, all of its outgoing links (in all layers) must be considered.
2. A random priority is assigned to each link $(i,j)^{\gamma}$. At each node i, a link with the highest priority is selected from all of the outgoing links of the node, and its correspondent node marked as visited.

A new random priority is assigned to each link for generating each new initial population, therefore the diversity of initial population could be insured.

7.2 Nondominated sorting and crowding distance

After calculating the multiplex path length of paths in population, a fitness must be assigned to each of these paths. NSGA-II assigns two values to each member of population to ensure the quality of solutions in terms of convergence and diversity[21] as follows:

1. ***Rank***: For the members of population, the nondominated solutions will have their *Rank* equal to 1. By excluding these solutions and finding nondominated solutions from the remaining set, the solutions with their *Rank* equal to 2 will be obtained. Continuing in this process will classify all solutions in the population, based on their nondomination level. Figure 9 shows an example of nondominated ranking in objective space for two objective functions. As can be seen, the solutions are ranked in three levels. Comparing two solutions, the one with lower *Rank* is a better efficient solution. However, in a case that both solutions have the same *Rank*, the *crowding distance* measure will determine the better solution.
2. ***Crowding Distance (CD)***: NSGA-II uses the distance of a solution i from its neighboring solutions ($i-1$ and $i+1$) in order to find the density of solutions. For a set of nondominated solutions with l members, crowding distance is calculated as follows:

$$CD = \begin{cases} \sum_{k=1}^{K} \frac{f_{i+1}^{k} - f_{i-1}^{k}}{f_{max}^{k} - f_{min}^{k}} & \text{if } i \in \{2,3,...,l-1\} \\ \infty & \text{else} \end{cases}$$

Where K is the number of objective functions. Figure 9 shows an example of calculating crowding distance for a solution in nondominated solutions of rank 3. In order to preserve the diversity of the solutions in a nondominated set, the solutions with high crowding distance should be selected for the next generations.

Hence, using these two values, the population will be sorted. For two solutions x and y, x is better than y if it belongs to the lower *Rank*, or they both belong to the same *Rank* but x has higher crowding distance (this relation called crowded-comparison operator).

7.3 Selection and Variation

NSGA-II uses the binary tournament selection procedure in order to reproduce individuals with the highest fitness. Each of the two selected solutions compared according to the crowded-comparison operator and the winners construct the mating pool. Afterward, the crossover and mutation operate on members of the mating pool.

For the *crossover* operator we used one-point crossover as follows:

1. Randomly select two parents (paths) from mating pool.
2. Randomly select a gene from one of the parents (ignoring layers tag).
3. Find the matching gene in the other parent. If there is no such gene, select two other parents until the mating pool is empty.
4. If a match is found, perform crossover with respect to layers tag (See Figure 8).
5. Detect and eliminate loops (for a loop repair function on shortest path problem in single-layer network refer to Ahn & Ramakrishna[22]).

For the *mutation* operator the following steps are performed:

1. Randomly select chromosome (path) from mating pool.
2. Randomly select a gene (node) from the selected chromosome (ignoring layers tag).
3. Create a new chromosome similar to the original one with a new random path (created based on the method used for generating initial population) starting from the selected node to the destination node and replace it with the original chromosome.
4. detect and eliminate loops.

7.4 Performance of NSGA-II

Suppose that $S = \{S_1, S_2, ..., S_i, ..., S_k\}$, be the set of k solution sets, each of them obtained by adjusting different values for NSGA-II parameters, and also suppose that S^* be the refrence solution set obtained by an exact algorithm. In order to evaluate different solution sets of S, we utilize three performance measures introduced by Ishibuhi *et al.*[23] (the reason for using more than one performance measure is the impossiblity of evaluating all aspect of resulting solutions with one performance measure). These measures include:

1. The number of members of the solution set (i.e., the cardinality of S_i) which denoted by $|S_i|$.

2. The ratio of nondominated solutions, denoted by $R_{NDS}(S_i)$ and calculated as follows:

$$R_{NDS}(S_i) = \frac{|S_i - \{x \in S_i \mid \exists m \in S^* : m \prec x\}|}{|S_i|} \tag{6}$$

3. Average distance to reference solution set, which calculated as follows:

$$D1_R = \frac{1}{|S^*|} \sum_{m \in S^*} min\{d_{xm} \mid x \in S_i\} \tag{7}$$

where d_{xm} is a distance between a solution in S_i and a solution in S^* as follows:

$$d_{xm} = \sqrt{(f_1^*(m) - f_1^*(x))^2 + \ldots + (f_L^*(m) - f_L^*(x))^2}$$

L is the number of objective functions (number of layers in our problem) and f^* means that the objective space is normalize based on the reference solution set.

It is obvious that a good approximate solution set, is the one with minimum value for $D1_R$, and maximum values for $|S_i|$ and $R_{NDS}(S_i)$.

In order to evaluate the performance of NSGA-II, first, we construct the S^* set by finding all of the influential Pareto paths on the Trade network of the year 2015 for any specific source and destination node. Afterward, we perform NSGA-II starting from the source node S to the destination node D, and calculated the three mentioned performance measures. We did this for any other combination of source and destination nodes and obtained the whole number of solutions ($\sum |S_i|$) and the average of $R_{NDS}(S_i)$ and $D1_R$ measures. Figure 10 shows the results for the performance of NSGA-II algorithm with different values for its parameters (i.e., population size, number of generation, crossover and mutation rates).

In order to explore the effect of different parameters on the performance of NSGA-II, we first fixed three of the parameters (population size$=40$, generation size$=50$, and mutation rate$=0.1$) and increased the crossover rate from 0 to 1. Figure 10(a) shows the effect of increasing crossover probability in the performance of NSGA-II based on three performance measures. As it can be seen, this increment causes improvement in the average ratio of nondominated solutions. However, this increment worsen the values for $\sum |S_i|$ and the average $D1_R$. Hence, in order to make a trade-off between these values, we set the crossover rate to 0.4 for the next steps. Then we vary the mutation rate between 0.02 and 1.6 to explore the effect of increasing the mutation rate in the performance of NSGA-II. Figure 10(b) shows that with increasing this probability, the average ratio of nondominated solutions improves gradually, and the total number of solutions remains stable. However, the average $D1_R$ parameters does not follow an speific pattern. This can be due to the search process which tend toward a random search in high mutation rate. Hence, we adjust the mutation rate to 0.08 for the next steps.

In order to explore the effect of generation size in performance of the NSGA-II, we set the value of other parameters based on the output of previous steps (i.e., crossover rate$=0.4$, mutation rate$=0.08$, and population size$=40$) and increased the generation size from 5 to 70 (see Figure 10(c)). The results show that the average $R_{NDS}(S_i)$ rises faster comparing with two previous parameters. But this increment makes the other two performance measures to perform worse. Hence, in order to achieve a good performance, we adjust the generation size equals to 30 for the next step.

Finally in order to see the effect of population size on performance of the NSGA-II, we set the value of other parameters based on the output of previous steps (generation size$=30$, mutation rate$=0.08$, and crossover rate$=0.4$) and changed the population size from 5 to 100. Based on our results, all of the

performane measures improves with increasing of this parameter. However, the average $D1_R$, is more sensitive to this parameter and improves quickly.

Hence based on our results, even with low value for the different parameters (in our case study, with generation size= 30, population size= 100, Crossover rate= 0.4, and Mutation rate= 0.08) the NSGA-II algorithm has a good performance at finding influential Pareto paths.

8 Supplementary Note 8 (Exact algorithm for finding multiobjective shortest path in multiplex network)

The basic idea behind this algorithm is traversing the network and constructing the search tree. The function $h(x)$ is a map from the nodes of the network and their position in the search tree. X holds the nodes of the search tree which have not been analyzed yet. Each node in search tree contains two values: the label of the corresponding node in network and the layer number of its parent link. For example $h(2) = [5,3]$ means that the node number 5 is in the second position of the corresponding search tree and

Algorithm 1 Multiplex multiobjective shortest path

Input: $\vec{G}'', S, D$
Output: influential Pareto paths from S to D

Initialization :

$Z_i \leftarrow \varnothing, \forall i \in V$
count$\leftarrow$ 1
h(count) $\leftarrow \{S, \varnothing\}$
$X = \{\text{count}\}$
$Z_S = path(1)$

Main Procedure:

while $X \neq \varnothing$ **do**
    x=lexicographically smallest element in X
    Remove x from X
    $[i \quad la] = h(x)$
    for $l \in L$ **do**
        for $(i,j)^l \in E_l$ **do**
            $path(count) = path(x) \oplus (i,j)^l$
            if $path(count)$ is nondominated in Z_j **then**
                $count = count + 1$
                add $(x, count)$ to ST
                $h(count) = [j,l]$
                $X = X \cup \{count\}$(in lexicographic order)
                $Z_j = Z_j \cup \{path(count)\}$
                Remove domnated paths from Z_j
                Remove correspondent tree nodes from X
            end if
        end for
    end for
end while
return Z_D ▷ set of nondominated paths from S to D

is accessible from its parent node using a link on the third layer. Z_i holds non-dominated paths from S to i. The function $path(x)$ returns a path from node S to node $h(x)$ and the operator $\oplus$ means the concatenation of two subpath. Despite the single objective case, when this algorithm first reaches the destination node, the algorithm cannot be terminated because of the possibility of finding a path which dominates the previous ones in the next iterations. Therefore it must be continued until the set X be empty. Hence in order to find the whole multiobjective shortest paths from node S to node D in multiplex network, the algorithm needs to compute the shortest paths from S to all the other nodes.

Supplementary References

1. Menichetti, G., Remondini, D., Panzarasa, P., Mondragón, R. J. & Bianconi, G. Weighted Multiplex Networks. *PLoS ONE* **9**, e97857+ (2014).
2. Dietz, L., Bickel, S. & Scheffer, T. Unsupervised prediction of citation influences. In *Proceedings of the 24th International Conference on Machine Learning*, ICML '07, 233–240 (ACM, New York, NY, USA, 2007).
3. Hangal, S., MacLean, D., Lam, M. S. & Heer, J. All friends are not equal: Using weights in social graphs to improve search. In *Workshop on Social Network Mining & Analysis, ACM KDD* (2010).
4. Liu, L., Tang, J., Han, J. & Yang, S. Learning influence from heterogeneous social networks. *Data Mining and Knowledge Discovery* **25**, 511–544 (2012).
5. Tzeng, G. & Huang, J. *Multiple Attribute Decision Making: Methods and Applications*. A Chapman & Hall book (Taylor & Francis, 2011).
6. Miettinen, K. *Nonlinear Multiobjective Optimization*. International Series in Operations Research & Management Science (Springer US, 2012).
7. Özlen, M. & Azizoğlu, M. Multi-objective integer programming: a general approach for generating all non-dominated solutions. *European Journal of Operational Research* **199**, 25–35 (2009).
8. Ehrgott, M. & Gandibleux, X. A survey and annotated bibliography of multiobjective combinatorial optimization. *OR-Spektrum* **22**, 425–460 (2000).
9. Teghem, J. Multi-objective combinatorial optimization. In Floudas, C. & Pardalos, P. (eds.) *Encyclopedia of Optimization*, 2437–2442 (Springer US, 2008), 2 edn.
10. Serafini, P. Some considerations about computational complexity for multi objective combinatorial problems. In Krabs, W. & Jahn, J. (eds.) *Recent advances and historical development of vector optimization*, 222–232 (Springer, 1987).
11. Magnani, M. & Rossi, L. Pareto Distance for Multi-layer Network Analysis. In Greenberg, A., Kennedy, W. & Bos, N. (eds.) *Social Computing, Behavioral-Cultural Modeling and Prediction*, vol. 7812 of *Lecture Notes in Computer Science*, 249–256 (Springer Berlin Heidelberg, 2013).
12. UN Comtrade Database. `http://comtrade.un.org`. Accessed: 2016-09-12.
13. Lee, K.-M. & Goh, K.-I. Strength of weak layers in cascading failures on multiplex networks: case of the international trade network. *Scientific reports* **6** (2016).
14. IMF World Economic Outlook Databases. `http://www.imf.org/external/data.htm`. Accessed: 2016-09-12.
15. Omodei, E., De Domenico, M. & Arenas, A. Characterizing interactions in online social networks during exceptional events. *arXiv preprint arXiv:1506.09115* (2015).

16. Sampson, S. F. *A novitiate in a period of change: An experimental and case study of social relationships* (Cornell University, 1968).

17. Tang, L., Wang, X. & Liu, H. Uncoverning groups via heterogeneous interaction analysis. In *2009 Ninth IEEE International Conference on Data Mining*, 503–512 (IEEE, 2009).

18. StarWars network. `https://github.com/evelinag/StarWars-social-network/tree/master/networks`. Accessed: 2016-09-03.

19. Magnani, M., Micenkova, B. & Rossi, L. Combinatorial analysis of multiple networks. *arXiv preprint arXiv:1303.4986* (2013).

20. Yu, H. & Lu, F. A multi-modal route planning approach with an improved genetic algorithm. *Adv. Geo-Spatial Inform. Sci* **38**, 193–202 (2012).

21. Coello, C. A. C., Dhaenens, C. & Jourdan, L. Multi-objective combinatorial optimization: Problematic and context. In *Advances in multi-objective nature inspired computing*, 1–21 (Springer, 2010).

22. Ahn, C. W. & Ramakrishna, R. S. A genetic algorithm for shortest path routing problem and the sizing of populations. *IEEE transactions on evolutionary computation* **6**, 566–579 (2002).

23. Ishibuchi, H., Yoshida, T. & Murata, T. Balance between genetic search and local search in memetic algorithms for multiobjective permutation flowshop scheduling. *IEEE transactions on evolutionary computation* **7**, 204–223 (2003).
